# Supplementary material for: scMitoMut for calling mitochondrial lineage-related mutations in single cells
Source: Brief Bioinform. 2025 Feb 26;26(1):bbaf072. doi: 10.1093/bib/bbaf072 (PMC11878546; doi:10.1093/bib/bbaf072)
Supplement: Supplementary_materials_bbaf072 [file supplementary_materials_bbaf072.pdf]

scMitoMut for calling mitochondrial lineage–related mutations in single cells

Wenjie Sun[1], Daphne van Ginneken[1], Leïla Perié[1]

Affiliations:

1. Institut Curie, Université PSL, Sorbonne Université, CNRS UMR168, Physique des Cellules et Cancer, 75005, Paris, France

Corresponding authors: sunwjie@gmail.com and leila.perie@curie.fr

Table of Contents

Supplementary Tables & Figures 2

Supplementary Table 1. Probability of multiple mutations per locus in a dataset. 2

Supplementary Table 2. Parameters in scMitoMut mutation calling. 2

Supplementary Table 3. Comparison between single cell mtDNA calling tools. 3

Supplementary Figure 1. Identifying lineage informative mtDNA mutation using a beta-binomial model 4

Supplementary Figure 2. Performance of the binomial-mixture distribution fitting in scMitoMut 5

Supplementary Figure 3. Performance of the beta-binomial model in scMitoMut 6

Supplementary Figure 4. Sensitivity of free parameters on the mutation calling using scMitoMut on single cell genome sequencing of cell line mixture dataset 7

Supplementary Figure 5. Benchmarking scMitoMut over SOTA with single cell genome sequencing of cell line mixture dataset 9

Supplementary Figure 6. scMitoMut separates epithelial and blood lineage in a CRC dataset 10

Supplementary Figure 7. scMitoMut separates epithelial and blood lineage in 10X PBMC dataset 11

Supplementary Figure 8. Benchmarking scMitoMut over SOTA methods using the PBMC 10X multiome dataset 12

Supplementary Figure 9. scMitoMut reveals lineage informative mitochondrial mutations in 10x Brain multiome dataset 12

Supplementary methods 13

Fit binomial mixture model with Expectation maximization (EM) algorithm 13

Fit beta binomial distribution with Maximum Likelihood Estimation (MLE) algorithm 14

Supplementary Tables & Figures

Mutation number	Probability of a specific locus has multiple mutant alleles
**100**	0.0021
**50**	0.0010
**25**	0.0005

Supplementary Table 1. Probability of multiple mutations per locus in a dataset.

We simulated the mutation events in the human mitochondrial genome (16k bp), assuming equal probability of mutations and calculated the probability of having multiple mutant alleles at a locus for different number of randomly samples mutations (25, 50, 100). The simulations were repeated 100000 times.

Parameters	Comment	Default value
**mean mtDNA depth per cell**	The cells that have equal or higher mean mtDNA depth will be selected for analysis	5
**binomial-mixture distribution classifier FDR threshold**	The cells with binomial-mixture distribution classifier FDR larger than the threshold will be selected for fitting the beta-binomial model	0.05
**Beta-binomial q-value threshold**	In a specific cell, if a locus has q-value smaller than the threshold will be called as mutant cell	0.01
**Clone size threshold**	If a mutation with clone size larger or equal than the threshold will be selected as lineage informative mutation	5

Supplementary Table 2. Parameters in scMitoMut mutation calling.

In this table, the free parameters of scMitoMut pipeline are listed, together with explanations and default values.

Feature	Signac	MQuad	Mitoclone2	scMitoMut
**Sequencing type**	scDNASeq<br>scATACSeq	scDNA<br>scATAC<br>RNAseq	scRNAseq	scDNAseq<br>scATAC
**Implementation**	R	Python	R	R (Rcpp)
**Mutation filtering**	VMR, reads-strand consistent threshold-based filtering	Binomial-mixture based statistical framework	Identify mutations unique to individuals	Beta-binomial distribution based statistical framework
**Single cell mutation calling**	AF threshold	NA	AF threshold	Beta-binomial distribution based statistical framework

Supplementary Table 3. Comparison between single cell mtDNA calling tools.

In this table we briefly compared the features between different single cell mtDNA calling tools. VMR: variant allele frequency variant mean ratio, AF: allele frequency.

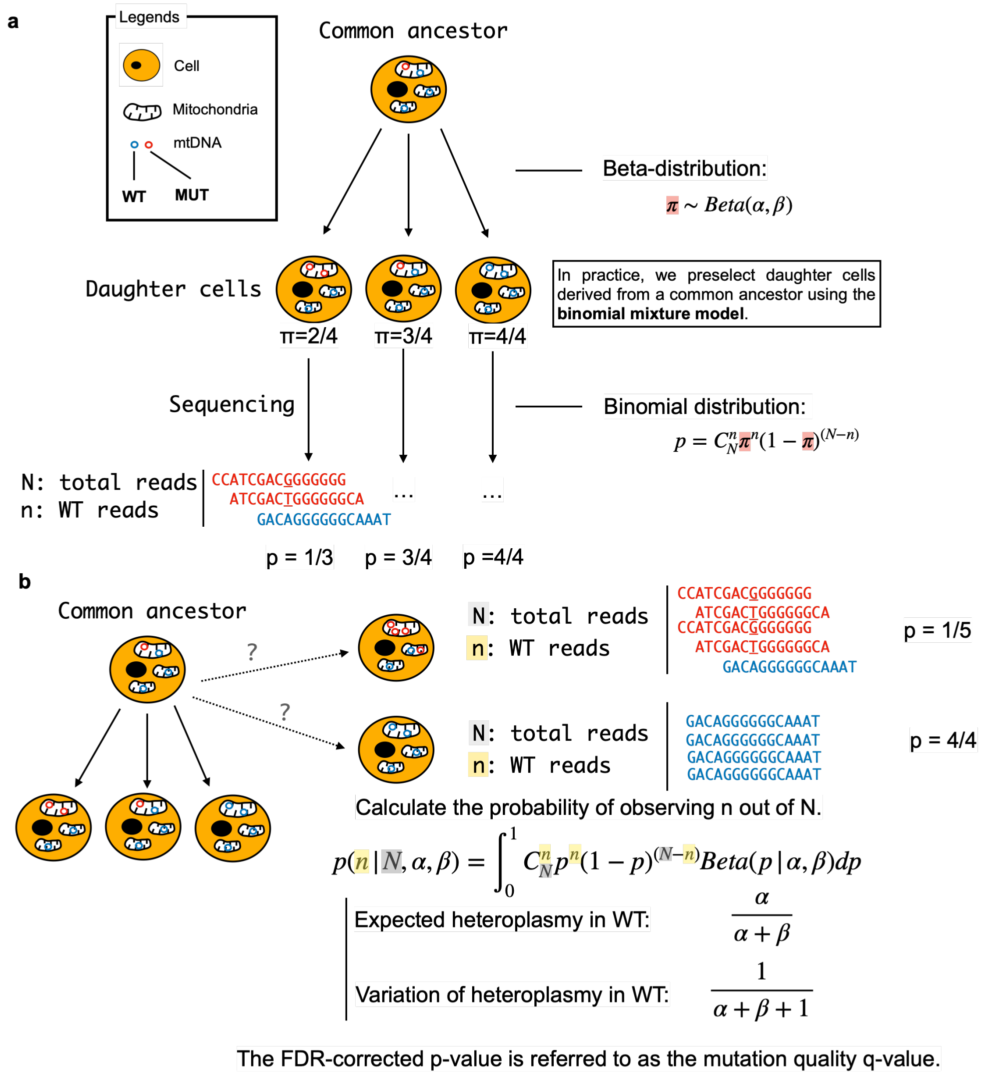

Supplementary Figure 1. Identifying lineage informative mtDNA mutation using a beta-binomial model

(a) Modeling single-cell mtDNA allele frequency using a beta-binomial distribution as implemented into scMitoMut, it corresponds to step 2 of Figure 1b. After sequencing, we obtained allele frequency by counting the reads. This frequency depends on the mtDNA heteroplasmy, mtDNA sampling, sequencing errors, and PCR errors and is modeled by a binomial process. The dynamic of mtDNA heteroplasmy ranges from 0 to 1 and depends on the dynamics of mtDNA as cells divide, this is modeled using a beta distribution. In the diagram, big yellow circles represent cells with black dot inside as nucleus. In each cell, there are multiple mitochondria and mtDNA. The small blue circles represent wild type mtDNA, while the red circles indicate mutant mtDNA. Both types are specific to a particular locus. It should be mentioned that the cells derived from common ancestors are unknown in practice and are preselected using binomial-mixture distribution.

(b) Assessing lineage-specific somatic mtDNA mutations per locus and per cell using the beta-binomial distribution, it corresponds to step 3 of Figure 1b. If cells come from a shared ancestor and are wild-type, they should follow the beta-binomial distribution fitted on wild-type cells of step 2 of Figure 1b, enabling us to calculate the probability of the cells being wild-type at a given locus, given its reads n at that locus and the total reads N. By adjusting the obtained probability with false discovery rate (FDR), the resulting adjusted probability acts as the mutation quality value, referred to as the q-value.

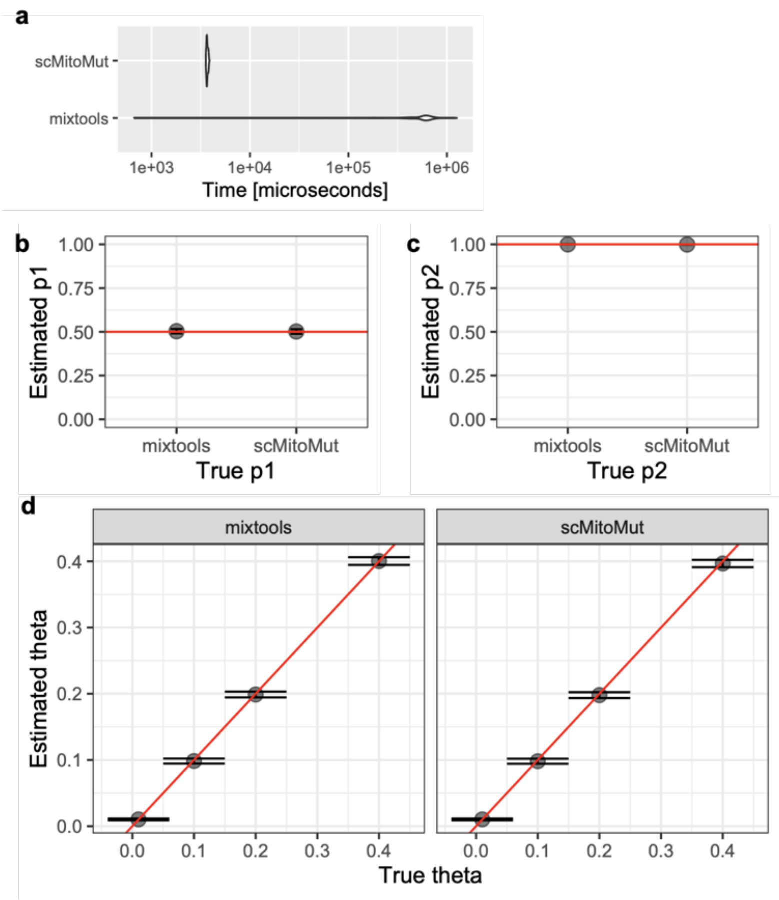

Supplementary Figure 2. Performance of the binomial-mixture distribution fitting in scMitoMut

(a) The time needed for model fitting of binomial-mixture distribution fitting for the R package mixtools and scMitoMut. The horizontal violin plot illustrates the distribution of time across 100 replications. The tested dataset is simulated binomial mixture distribution with two binomial distributions with $\theta = 0.5$, $\pi1 = 0.992$ and $\pi2 = 1$ respectively. The n follows a log-normal distribution (log-mean = 2 and log-SD = 1) rounded up to the nearest non-negative integer. Each simulation includes 1000 observations.

(b), (c) The fitting accuracy of parameters π_1 (p1) and π_2 (p2) obtained using the R package mixtools and scMitoMut. The red line indicates the true value.

(d) The fitting accuracy for various values of parameter obtained using the R package mixtools and scMitoMut. The x and y axes represent the true and estimated values respectively, with the red line denoting the y=x diagonal. For fitting accuracy, the simulations follow this design: $\pi_1 = 0.5, \pi_2 = 1, \theta = 0.01, 0.1, 0.2, 0.4$ and n following a log-normal distribution (log-mean=2, log-SD=1) rounded to the nearest non-negative integer. Each simulation contains 5000 observations. Error bars in (b)–(d) denote the mean ± SD.

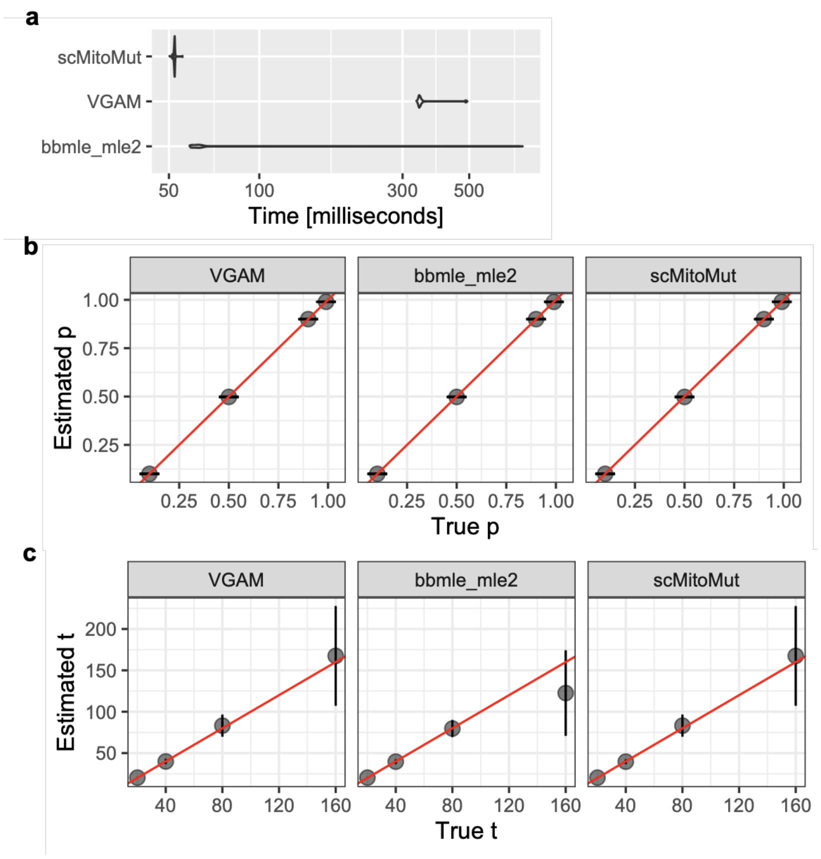

Supplementary Figure 3. Performance of the beta-binomial model in scMitoMut

(a) The beta-binomial model fitting speed between the R package VGAM, bbmle and scMitoMut. The horizontal violin plot illustrates the distribution of time across 100 replications. The BBD dataset was simulated with a mean probability parameter θ of 0.5, and a super-dispersion parameter ϕ of 20. Each simulation includes 5000 observations.

(b), (c) The fitting accuracy for parameter θ and ϕ using the R packages VGAM, bbmle, and scMitoMut. The red line denotes y = x diagonal. The parameter values are: mean probability parameter θ of 0.1, 0.5, 0.9, and 0.99; dispersion parameter ϕ of 20, 40, 80, 160. In the simulation, n adheres to a log-normal distribution with a log-mean of 2 and log-SD of 1, subsequently rounded up to the nearest non-negative integer. Each simulation encompasses 5000 observations. The error bars in panels B and C represent the mean ± SD.

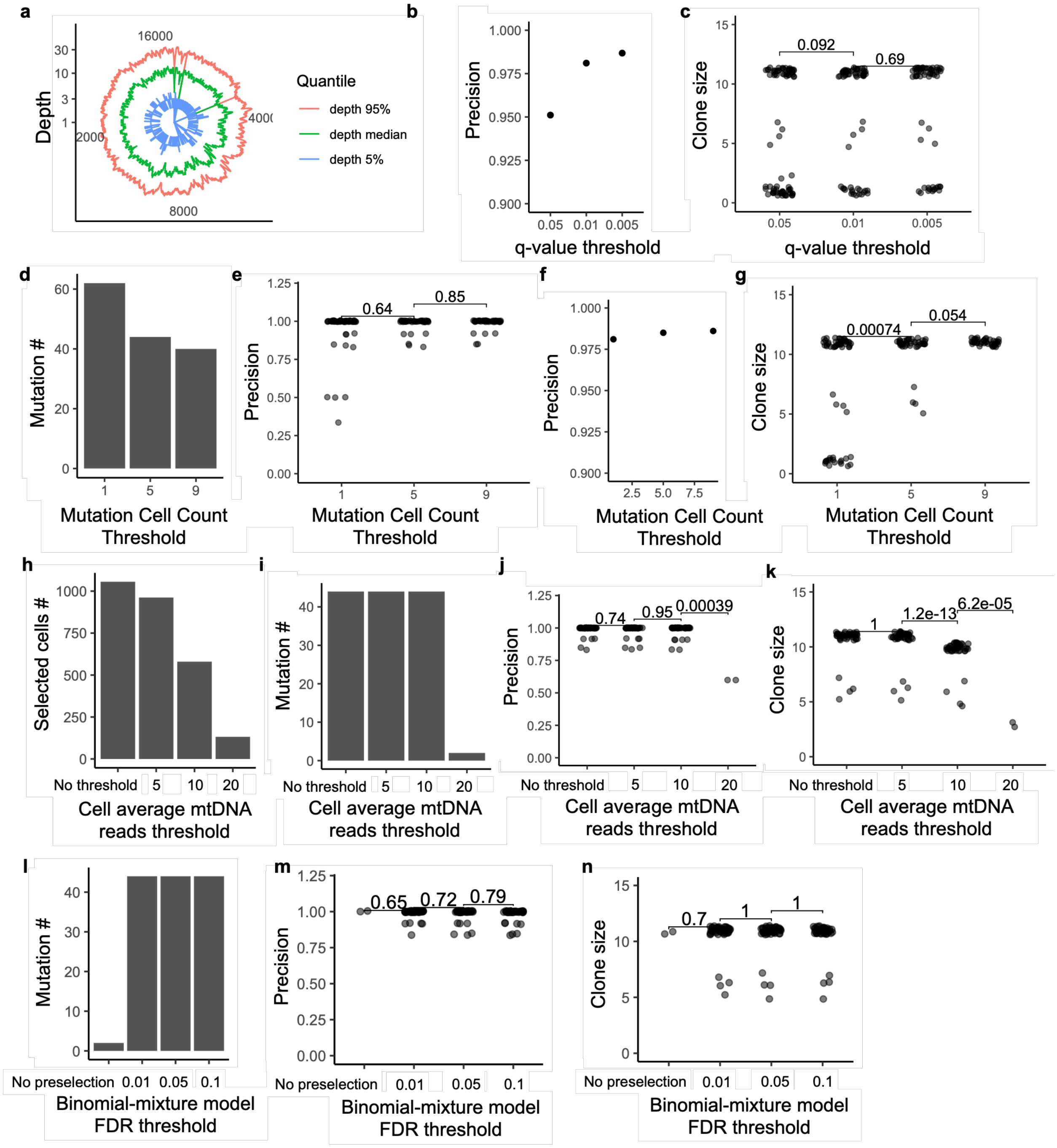

Supplementary Figure 4. Sensitivity of free parameters on the mutation calling using scMitoMut on single cell genome sequencing of cell line mixture dataset

(a) Polar plot displaying the quantile of mtDNA sequencing depth in the 10X Genomics single-cell genome sequencing dataset. Mitochondrial locus is shown by angle and sequence depth by radius. Blue, green, and pink indicate the 5th, 50th, and 95th percentiles, respectively, of depth across the population. All cells were analysed with no filtering.

(b) Clone size weighted average precision of mutation calling with different q-value thresholds. The precision is calculated by dividing the number of MKN45 mutant cells by the total number of mutant cells, resulting in a clone size weighted average precision per mutation.

(c) The number of cells per mutation (clone size) for different q-value thresholds. The n is 79, 62 and 59 for q-value threshold of 0.05, 0.01 and 0.005 respectively.

(d) Number of mutations detected with different thresholds of mutant cell numbers.

(e) Clone size weighted average precision of mutations with different thresholds of mutant cell numbers. The sample sizes are 64, 44, and 40 for clone size thresholds of 1, 5, and 9, respectively.

(f) Precision of mutation calling with different thresholds of mutant cell numbers. The precision is calculated by dividing the number of MKN45 mutant cells by the total number of mutant cells, resulting in a clone size weighted average precision per mutation.

(g) Number of cells per mutation for different mutant cell number thresholds. The sample sizes are 64, 44, and 40 for clone size thresholds of 1, 5, and 9, respectively.

(h) Cell number for different mean mtDNA depth per cell thresholds.

(i) Number of detected mutations for different mean mtDNA depth per cell thresholds.

(j-k) Precision (j) and clone size (k) of mutation calling with different mean mtDNA depth per cell thresholds. The sample sizes are 44, 44, 44, and 2 for different mean mtDNA depth per cell thresholds respectively: no threshold, 5, 10, and 20 respectively.

(l) Number of detected mutations for different binomial-mixture distribution classifier FDR thresholds.

(m-n) Precision (m) and clone size (n) of mutation calling for different binomial-mixture distribution classifier FDR thresholds. The sample sizes are 2, 44, 44, and 44 for different beta-binomial model FDR threshold respectively: no threshold, 0.01, 0.05, and 0.1.

In all the above analysis, only mutations with at least one MKN45 cell passing the threshold were included in the statistics. For (b-g), the average coverage threshold of mtDNA per cell was 5, and the beta-binomial model FDR threshold was set to 0.05. For (d-g), mutant cells were initially filtered using a q-value < 0.01. For (i-n), mutations were called with q-value threshold of 0.01 and clone size threshold of 5. In (c), (e), (g), (j), (k), (m) and (n), each dot represents a mutation, the Two-sided Wilcoxon Test was used to compare mutation precision, with the *p*-value displayed at the top.

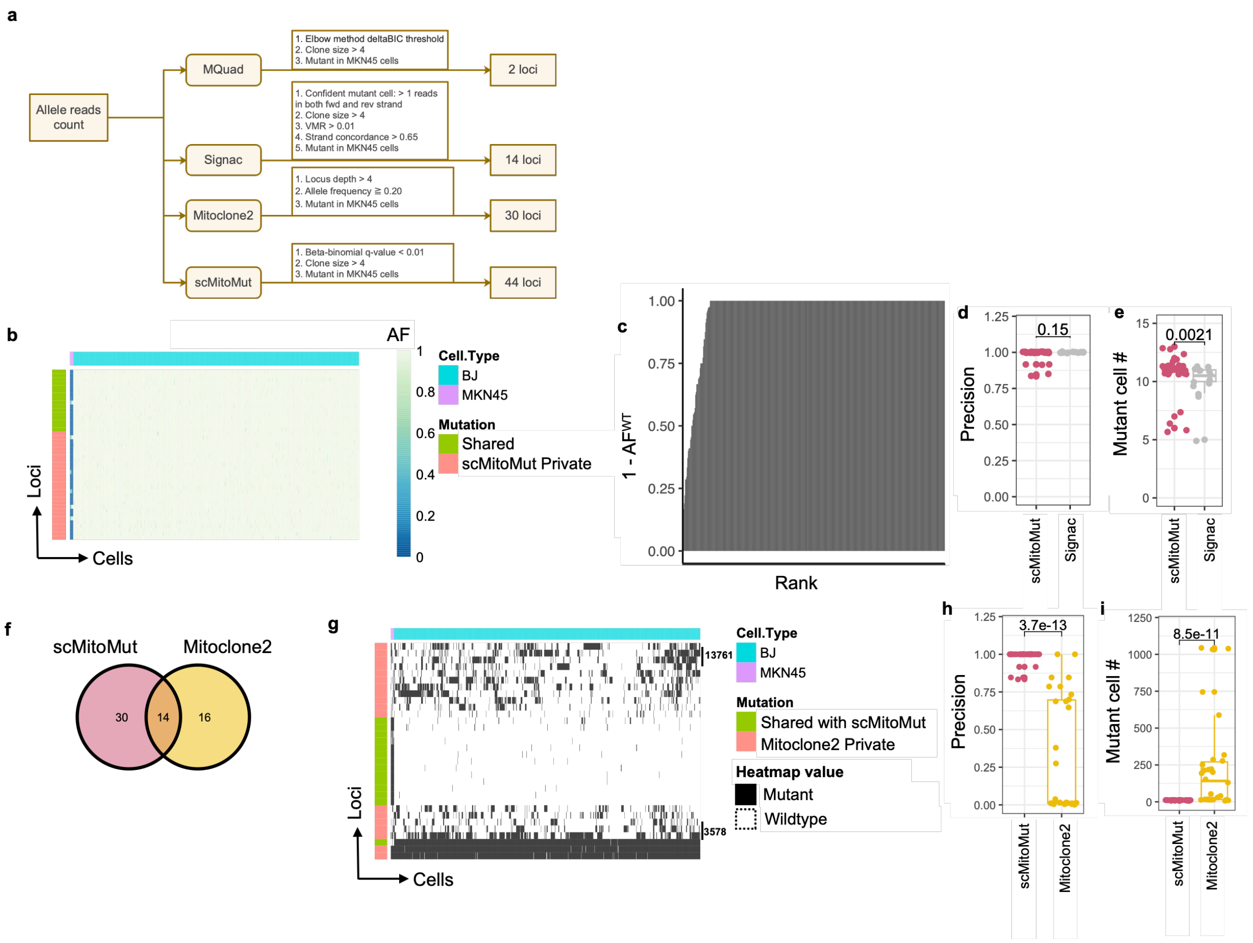

Supplementary Figure 5. Benchmarking scMitoMut over SOTA with single cell genome sequencing of cell line mixture dataset

(a) Parameters used for scMitoMut, Signac, MQuad, Mitoclone2. For scMitoMut, mutant cells are identified when the q-value < 0.01 and mutant cells >= 5. In the Signac pipeline, we followed default settings in its vignette: the informative locus should have a variant mean ratio (VMR) > 0.1; strand correlation > 0.65; confident mutant cells >= 5 (confident mutant cell is defined by having at least 2 mutant reads in forward and reverse strand reads respectively). For MQuad, we used the auto-determined delta Bayesian Information Criterion (deltaBIC) threshold to filter mutations. For Mitoclone2, we applied the thresholds of locus depth > 4 and allele frequency >= 0.20.

(b) The heatmap shows allele frequency of mutations per cell (columns) and per locus (rows). Blue shading represents wild type allele frequency. An annotation bar above the heatmap indicates the cell type (purple for MKN45 cells, and blue for BJ cells). Another annotation bar on the right shows mutations exclusively identified by scMitoMut, in red, and shared mutations between scMitoMut with MQuad and Signac, in green.

(c) Mutation allele frequency distribution for a total of 464 mutant events identified in MKN45 cells, with the (1 - wild-type allele frequency) displayed in ascending order.

(d) The scatter plot shows the lineage precision of scMitoMut and Signac results. The mutations with at least one MKN45 cell were analysed. Each dot in the scatter plot represents a mutation; Precision was calculated as the ratio of mutant MKN45 to total mutant cells for each mutation. n = 44 mutations in scMitoMut and n = 14 mutations in Signac.

(e) The scatter plot showing the mutant cell number for each mutation with scMitoMut and Signac. The mutations with at least one MKN45 cell are analysed. n = 44 mutations in scMitoMut and n = 14 mutations in Signac.

(f) Number of mutations (locus) identified by the scMitoMut (red) and Mitoclone2 (yellow) using the parameters described in (a).

(g) The heatmap shows mutation binary status of mutations per cell (columns) and per locus (rows). White presents wild type, and black represents mutant. An annotation bar above the heatmap indicates the cell type (purple for MKN45 cells, and blue for BJ cells). Another annotation bar on the right shows mutations exclusively identified by Mitoclone2, in red, and shared mutations between scMitoMut and Mitoclone2, in green. The locus 13761 and 3578 each has two mutant alleles.

(h) and (i), the lineage precision (g) and mutant cell number per mutations with scMitoMut and Mitoclone2. The mutations with at least one MKN45 cell were analysed. Each dot in the scatter plot represents a mutation; Precision was calculated as the ratio of mutant MKN45 to total mutant cells for each mutation. n = 44 for scMitoMut and n = 32 for Mitoclone2.

Statistical analysis was conducted using the two-sided Wilcoxon Test, with p-values indicated in the plot.

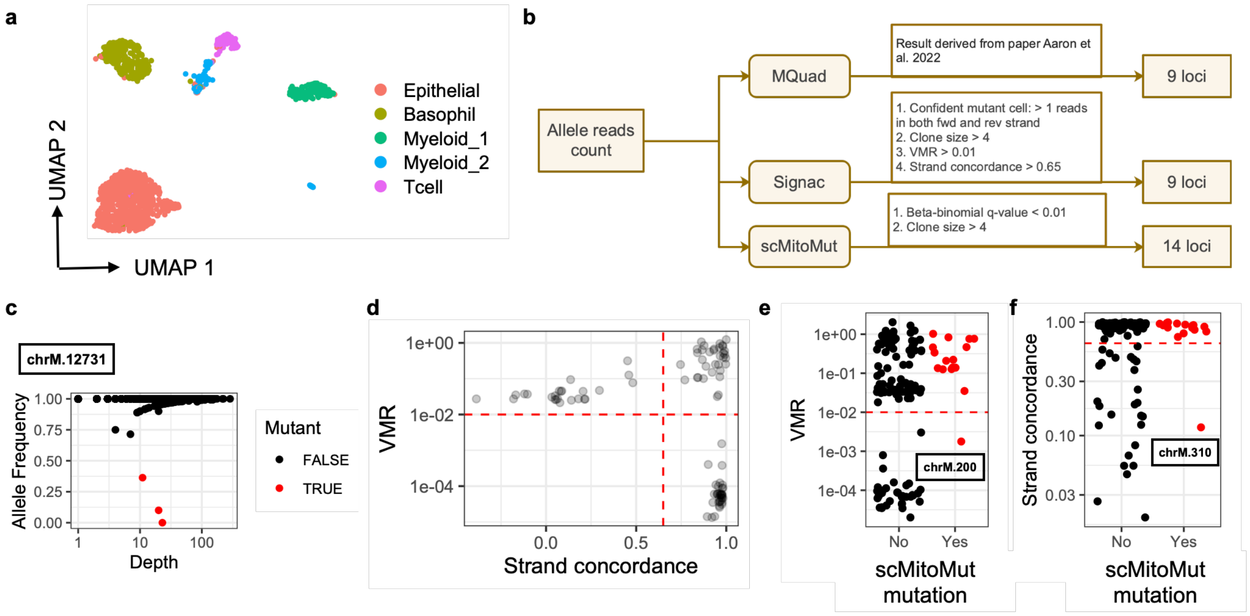

Supplementary Figure 6. scMitoMut separates epithelial and blood lineage in a CRC dataset

(a) UMAP representation of immune and cancer epithelial cells from the CRC dataset, colored by cell type using Seurat with default parameters.

(b) Parameters used for scMitoMut, Signac and MQuad. For scMitoMut, mutant cells are called if the q-value < 0.01 and mutant cells >= 5. In the Signac pipeline, we followed default settings in its vignette: the informative locus should have a Variant Mean Ratio (VMR) > 0.1; strand correlation > 0.65; confident mutant cells >= 5 (confident mutant cell is defined by having at least 2 mutant reads in forward and reverse strand reads respectively). For MQuad, we used the auto-determined delta Bayesian Information Criterion (deltaBIC) threshold to filter mutations.

(c) Sequencing depth and allele frequency of locus chrM.12731. Each dot represents a cell, with cells having scMitoMut mutation q-value < 0.01 highlighted in red.

(d) Variant mean ratio (VMR) and strand correlation distribution from the Signac result. Each dot is a mutation. Only mutations with at least 5 confident mutant cells are included in the plot. Each dot represents a mutation locus, and red lines indicate the default thresholds in Signac for VMR (0.01) and strand correlation (0.65).

(e) and (f) Signac VMR or strand concordance profiling with scMitoMut mutations called by: q-value < 0.05 and at least 5 mutant cells. Each dot is a locus. The x-axis shows the mutation status identified by scMitoMut, and the y-axis is the VMR value of the mutation (e) or strand concordance of mutation reads (f). The red line represents the threshold used by Signac which is 0.01 for VMR 0.01 and 0.65 for strand concordance 0.65.

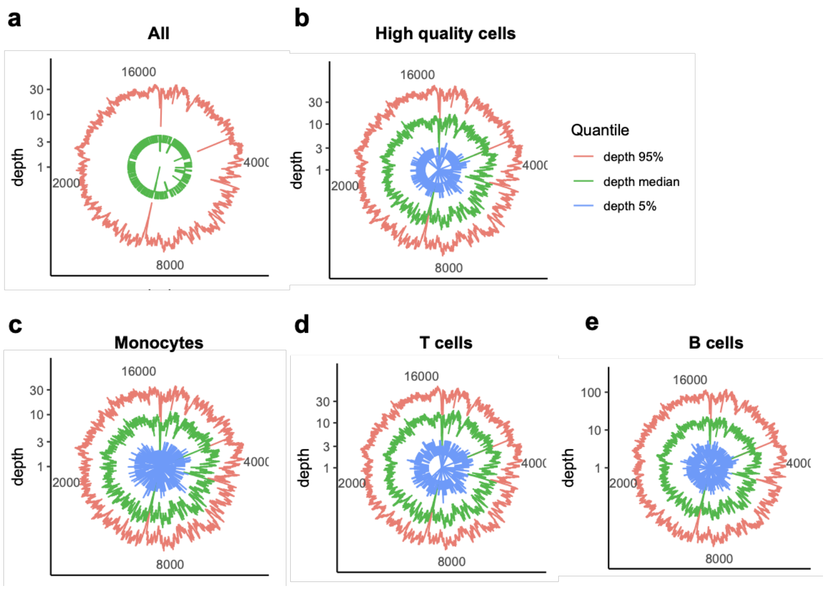

Supplementary Figure 7. scMitoMut separates epithelial and blood lineage in 10X PBMC dataset

(a) Polar plot displaying the quantile of mtDNA sequencing in the 10X PBMC multiome dataset scATACSeq unfiltered cells. Mitochondrial locus is shown by angle and sequence depth by radius. Blue, green, and pink indicate the 5th, 50th, and 95th percentiles, respectively, of depth across the population.

(b) Same as (a) but for high quality cells, which have a median mtDNA sequencing depth greater than 5.

(c-e) Same as (a) but for monocytes (c), T cells (d) and B cells (e) mtDNA sequencing depths among the high-quality cells of (b).

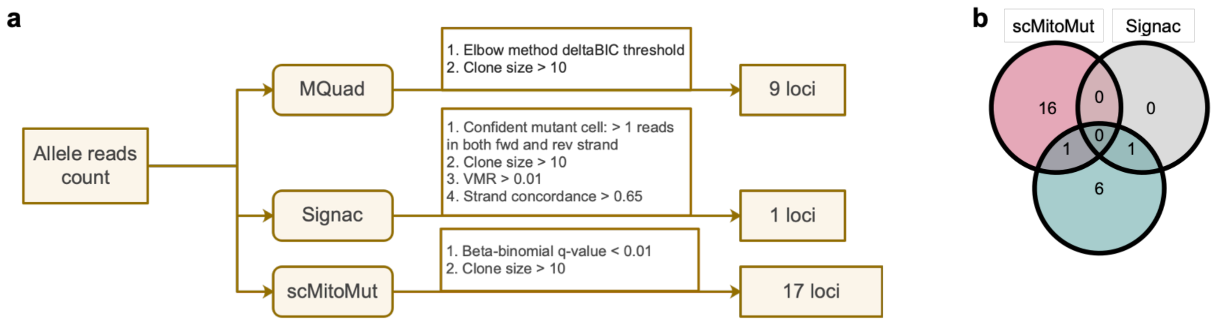

Supplementary Figure 8. Benchmarking scMitoMut over SOTA methods using the PBMC 10X multiome dataset

(a) Parameters used for scMitoMut, Signac and MQuad. For scMitoMut, mutant cells are identified when the q-value < 0.01 and mutant cells > 10. In the Signac pipeline, we followed default settings in its vignette: the informative locus should have a Variant Mean Ratio (VMR) > 0.1; strand correlation > 0.65; confident mutant cells >= 5 (confident mutant cell is defined by having at least 2 mutant reads in forward and reverse strand reads respectively). For MQuad, we used the auto-determined delta Bayesian Information Criterion (deltaBIC) threshold to filter mutations.

(b) Number of mutations identified by the three tools: scMitoMut in red, Signac in grey, and MQuad in green using the parameters in (a).

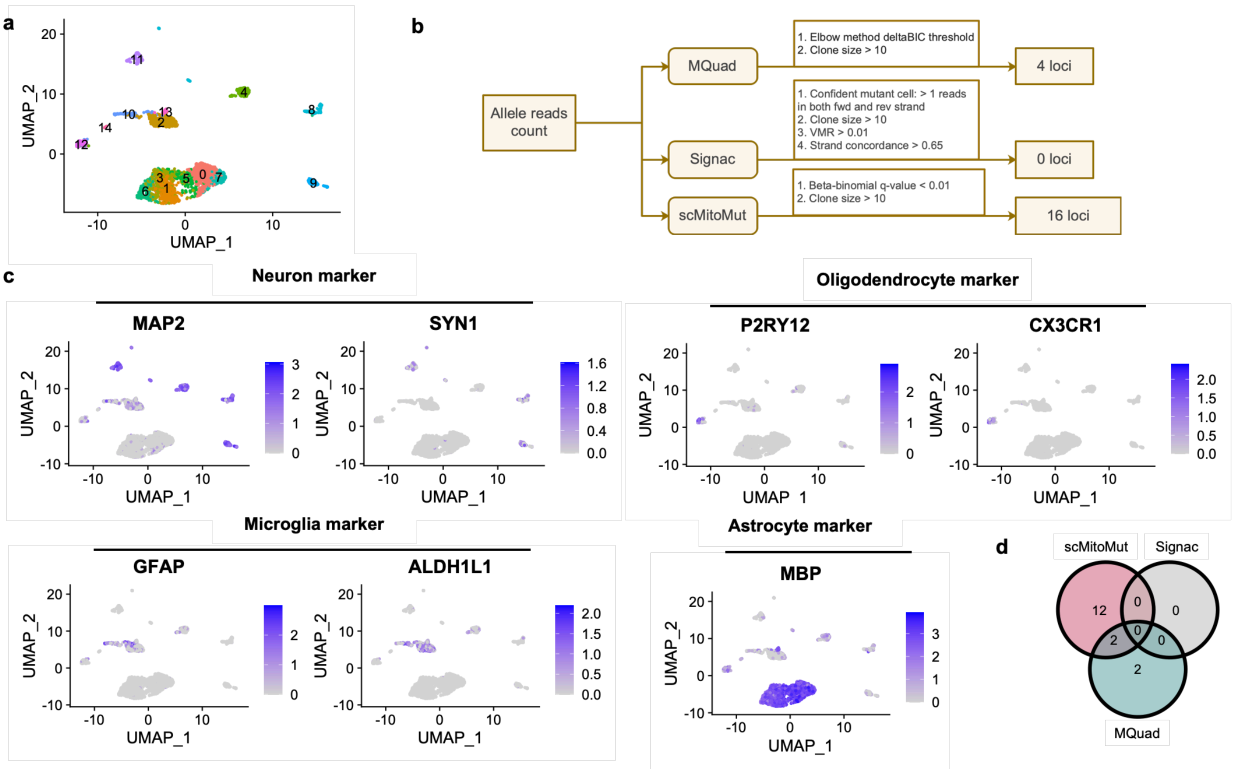

Supplementary Figure 9. scMitoMut reveals lineage informative mitochondrial mutations in 10x Brain multiome dataset

(a) UMAP of the brain 10X multiome dataset. The clusters identified by RNA are labelled by cluster id and annotated by colours using Seurat with default parameters.

(b) Parameters used for scMitoMut, Signac and MQuad. For scMitoMut, mutant cells are identified with the q-value < 0.01 and mutant cells > 10. In the Signac pipeline, we followed default settings in its vignette: the informative locus should have a Variant Mean Ratio (VMR) > 0.1; strand correlation > 0.65; confident mutant cells > 10 (confident mutant cell is defined by having more than two mutant reads in forward and reverse strand reads respectively). For MQuad, we used the auto-determined delta Bayesian Information Criterion (deltaBIC) threshold to filter mutations and clone size > 10.

(c) The same UMAP as in (a) but color-coded for the expression of gene markers of the neuron, astrocyte, microglia and oligodendrocyte.

(d) Number of mutations identified by the three tools: scMitoMut in red, Signac in grey, and MQuad in green using the parameter in (b).

Supplementary methods

Fit binomial mixture model with Expectation maximization (EM) algorithm

For a binomial mixture distribution (1) with 2 components $k = [1,2]$ representing two statuses: wild type and mutant. The binomial mixture distribution has parameters $\Theta = [\pi_1, \theta_1, \pi_2, \theta_2]$, for specific observation i with success event number n_i and total event number N_i.

$$P(n_i|N_i, \Theta) = \pi_1 Binom(n_i|N_i, \theta_1) + \pi_2 Binom(n_i|N_i, \theta_2) \qquad (1)$$

We fit the model by expectation maximization algorithm.

Initialization: Start by randomly initializing the parameters of the two binomial distributions Θ, and assign it to Θ_{old}.

Expectation step (E-step): Given the current parameters Θ_{old}, calculate the probability (2) that each cell come from the binomial distribution k.

$$P(z_i = k|n_i, \Theta_{old}) = [\Sigma_{l=1}^{2} \frac{\pi_{l,old}}{\pi_{k,old}} (\frac{\theta_{l,old}}{\theta_{k,old}})^{n_i} (\frac{1-\theta_{l,old}}{1-\theta_{k,old}})^{N_i - n_i}]^{-1} \qquad (2)$$

Maximization step (M-step): Update the parameters to have new Θ.

$$\pi_k = \frac{1}{S} \Sigma_{i=1}^{S} P(z_i = k|n_i, \Theta_{old}) \qquad (3)$$

$$\theta_k = \frac{\Sigma_{i=1}^{S} n_i P(z_i = k|n_i, \Theta_{old})}{\Sigma_{j=1}^{S} N_j P(z_j = k|n_j, \Theta_{old})} \qquad (4)$$

In (3) and (4), S is the cell number.

Iteration: Assign the updated Θ to Θ_{old}, and repeat E-step and M-step until the log likelihood function (5) converges.

$$lnf(\vec{n}, \vec{N}) = \Sigma_{i=1}^{S} lnP(n_i|N_i, \Theta) = \Sigma_{i=1}^{S} ln(\pi_1 Binom(n_i|N_i, \theta_1) + \pi_2 Binom(n_i|N_i, \theta_2)) \qquad (5)$$

Note: The EM algorithm was adapted from a Medium blog.: https://jingluan-xw.medium.com/binomial-mixture-model-with-expectation-maximum-em-algorithm-feeaf0598b60.

Fit beta binomial distribution with Maximum Likelihood Estimation (MLE) algorithm

For the beta-binomial distribution (6) with parameter α and β.

$$P(n_i|N_i,\alpha,\beta) = \binom{N_i}{n_i}\frac{\Gamma(\alpha+\beta)}{\Gamma(\alpha)\Gamma\beta}\Gamma(\alpha+n_i)\frac{\Gamma(\beta+N_i-n_i)}{\Gamma(\alpha+\beta+N_i)} \qquad (6)$$

We use MLE algorithm to fit beta-binomial distribution to S observations (here are S cells), for a specific observation i with success event number n_i and total event number N_i.

Firstly, we define the likelihood (7) giving parameters α and β.

$$f(\vec{n},\vec{N}) = \Pi_{i=1}^{S}(\binom{N_i}{n_i}\frac{\Gamma(\alpha+\beta)}{\Gamma\alpha\Gamma\beta}\Gamma(\alpha+n_i)\frac{\Gamma(\beta+N_i-n_i))}{\Gamma(\alpha+\beta+N_i)} \qquad (7)$$

From (7), derived the log likelihood function (8).

$$lnf(\vec{n},\vec{N}) = \Sigma_{i=1}^{S}ln(\binom{N_i}{n_i}\frac{\Gamma(\alpha+\beta)}{\Gamma\alpha\Gamma\beta\Gamma(\alpha+\beta+N_i)}\Gamma(\alpha+n_i)\Gamma(\beta+N_i-n_i))$$

$$= S[ln\Gamma(\alpha+\beta) - ln\Gamma(\alpha) - ln\Gamma(\beta)] - \Sigma_{i=1}^{S}ln\Gamma(\alpha+\beta+N_i) + \Sigma_{i=1}^{S}ln\binom{N_i}{n_i} + \Sigma_{i=1}^{S}ln\Gamma(\alpha$$

$$+ n_i) + \Sigma_{i=1}^{S}ln\Gamma(\beta+N_i-n_i) \qquad (8)$$

After beeing initialized, the parameter α and β are fitted using Newton-Raphson method by maximizing the likelihood.

$$[\alpha\ \beta\ ]_{New} = [\alpha\ \beta\ ]_{old} - J^{-1}([\alpha\ \beta\ ]_{old})\nabla f([\alpha\ \beta\ ]_{old}) \qquad (9)$$

In equation (9), the Jacobian matrix J is defined as (10).

$$J = \begin{bmatrix}\frac{\partial}{\partial\alpha^2}lnf(\vec{n},\vec{N}) & \frac{\partial}{\partial\alpha\partial\beta}lnf(\vec{n},\vec{N}) & \frac{\partial}{\partial\alpha\partial\beta}lnf(\vec{n},\vec{N}) & \frac{\partial}{\partial\beta^2}lnf(\vec{n},\vec{N})\end{bmatrix} \qquad (10)$$

The equations (11), (12), (13), (14), and (15) define the partial derivatives with respect to α and/or β in the log-likelihood function, that are used in equation (9) and (10).

$$\frac{\partial}{\partial\alpha}lnf(\vec{n},\vec{N}) = S[\gamma_0(\alpha+\beta) - \gamma_0(\alpha)] - \Sigma_{i=1}^{S}\gamma_0(\alpha+\beta+N_i) + \Sigma_{i=1}^{S}\gamma_0(\alpha+n_i) \qquad (11)$$

$$\frac{\partial}{\partial\beta}lnf(\vec{n},\vec{N}) = S[\gamma_0(\alpha+\beta) - \gamma_0(\beta)] - \Sigma_{i=1}^{S}\gamma_0(\alpha+\beta+N_i) + \Sigma_{i=1}^{S}\gamma_0(\beta+N_i-n_i) \qquad (12)$$

$$\frac{\partial}{\partial\alpha^2}lnf(\vec{n},\vec{N}) = S[\gamma_1(\alpha+\beta) - \gamma_1(\alpha)] - \Sigma_{i=1}^{S}\gamma_1(\alpha+\beta+N_i) + \Sigma_{i=1}^{S}\gamma_1(\alpha+n_1) \qquad (13)$$

Supplementary materials

$$\frac{\partial}{\partial\beta^2} lnf(\vec{n},\vec{N}) = S[\gamma_1(\alpha+\beta) - \gamma_1(\beta)] - \Sigma_{i=1}^{S}\gamma_1(\alpha+\beta+N_i) + \Sigma_{i=1}^{S}\gamma_1(\beta+N_i-n_i) \qquad (14)$$

$$\frac{\partial}{\partial\alpha\partial\beta} lnf(\vec{n},\vec{N}) = N\gamma_1(\alpha+\beta) - \Sigma_{i=1}^{N}\gamma_1(\alpha+\beta+n_i) \qquad (15)$$

Note, the Maximum Likelihood Estimation (MLE) algorithm was adapted from video of YouTube channel "statisticsmatt":

https://www.youtube.com/watch?v=TKkB4_1kQNQ&list=PLDJYmoOMUVQVxPlTm00IN7XRkWdwJeyIz&index=3
